# Anti-Myeloperoxidase (MPO)-Positive Granulomatosis With Polyangiitis Presenting With Pulmonary and Cutaneous Vasculitic Flares in End-Stage Renal Disease: A Diagnostic and Therapeutic Challenge

**DOI:** 10.7759/cureus.102954

**Published:** 2026-02-04

**Authors:** Yash Ranga, Riddhi Agarwal, Bryan Ashong

**Affiliations:** 1 Internal Medicine, Jawaharlal Nehru Medical College, Ajmer, Ajmer, IND; 2 Internal Medicine, Nil Ratan Sircar Medical College and Hospital, Kolkata, IND; 3 Anesthesiology, University of South Florida Morsani College of Medicine, Tampa, USA

**Keywords:** anti-mpo granulomatosis with polyangiitis, atypical gpa flare, cutaneous vasculitis, end-stage renal disease, multi-organ involvement, negative bronchoalveolar lavage, p-anca positive vasculitis, pulmonary vasculitis, rituximab induction therapy, systemic vasculitis

## Abstract

Granulomatosis with polyangiitis (GPA) is a systemic small-vessel vasculitis typically associated with anti-proteinase-3 antibodies. Anti-myeloperoxidase (MPO) positivity is uncommon in GPA and often creates clinical overlap with microscopic polyangiitis. Managing atypical flares in patients with end-stage renal disease (ESRD) is particularly complex, as traditional goals such as renal recovery are absent, shifting the focus to life-threatening extrarenal manifestations. We present the case of a 50-year-old woman with known anti-MPO-positive GPA and ESRD on peritoneal dialysis who presented with progressive dyspnea, hemoptysis, fever, and painful cutaneous blistering on the extremities. Vital signs were notable for fever and mild tachypnea. Laboratory evaluation revealed a creatinine of 11.6 mg/dL, blood urea nitrogen of 55 mg/dL, elevated anti-MPO titers (2.3 IU/mL; normal 0-0.9), and a perinuclear antineutrophil cytoplasmic antibody titer of 1:80. Imaging demonstrated bilateral multifocal ground-glass opacities, tree-in-bud nodules, and scattered pulmonary nodules. Notably, bronchoscopy with bronchoalveolar lavage (BAL) was negative for diffuse alveolar hemorrhage and infection, which could have delayed the diagnosis. However, a biopsy of the cutaneous lesions confirmed leukocytoclastic vasculitis. Based on the integration of serologic, radiologic, and histopathologic findings, a systemic vasculitic flare was diagnosed. The patient was treated with pulse-dose methylprednisolone followed by rituximab induction (375 mg/m² weekly × 4). Cyclophosphamide was intentionally avoided due to her advanced renal disease and side effect profile. She achieved rapid clinical remission, with complete resolution of hemoptysis and healing of skin lesions. This case illustrates that severe MPO-positive GPA can manifest even in dialysis-dependent patients. A key clinical point is that negative BAL findings do not exclude active pulmonary vasculitis, necessitating a multimodal diagnostic approach. Furthermore, rituximab represents a safe and effective induction strategy in high-risk patients where renal salvage is no longer the objective, emphasizing the need for highly individualized management in atypical vasculitic presentations.

## Introduction

Granulomatosis with polyangiitis (GPA) is an autoimmune vasculitic disorder with a global prevalence of 20-160 cases per million and an incidence of 0.5-20 cases per million per year [1]. It typically involves the sinuses, nasal mucosa, lungs, kidneys, and skin. Antibody testing usually reveals anti-proteinase-3 (PR3) antibodies (formerly cytoplasmic antineutrophil cytoplasmic antibody (ANCA)) in approximately 80-90% of cases [1]. Anti-myeloperoxidase (MPO) antibodies are positive in only about 5-10% of GPA cases [2].

We present an unusual case of anti-MPO antibody-positive GPA presenting with a pulmonary and cutaneous vasculitis flare. Although GPA flares are well described, management in patients with end-stage renal disease (ESRD) is underrepresented, particularly when renal recovery is no longer the goal. This case highlights successful individualized induction therapy with rituximab in place of cyclophosphamide due to toxicity concerns and underscores the evolving phenotypic overlap between GPA and microscopic polyangiitis (MPA).

## Case presentation

**Figure 1:**
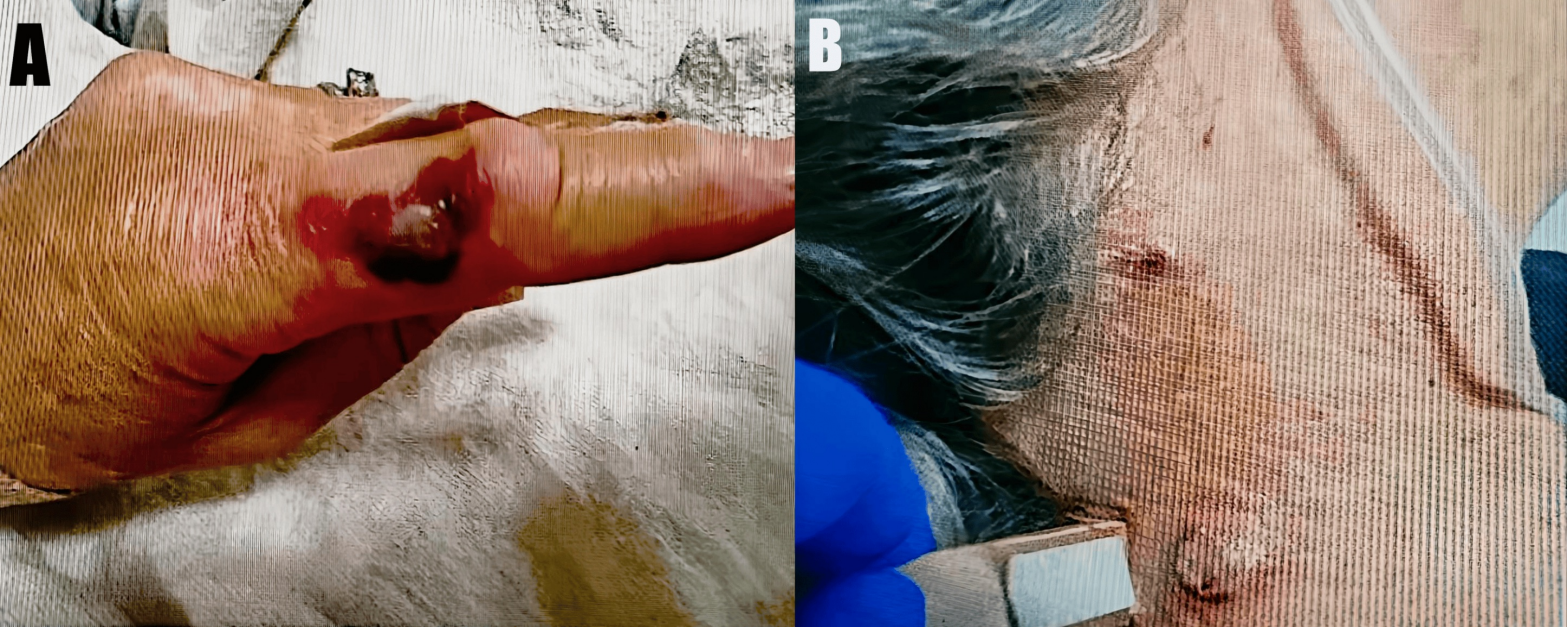
Painful cutaneous blistering lesions on the finger (a) and neck (b)

A 50-year-old woman with a past medical history of anti-MPO-positive GPA, noncompliance with steroid therapy, hypertension, and ESRD on peritoneal dialysis presented to the ED with progressive shortness of breath, hemoptysis, fatigue, subjective fever, and painful blistering lesions involving the fingers, elbows, and lower extremities (Figure 1), along with minimal bilateral lower-extremity swelling. She was comanaged by internal medicine, pulmonology, rheumatology, nephrology, and dermatology teams.

**Figure 2:**
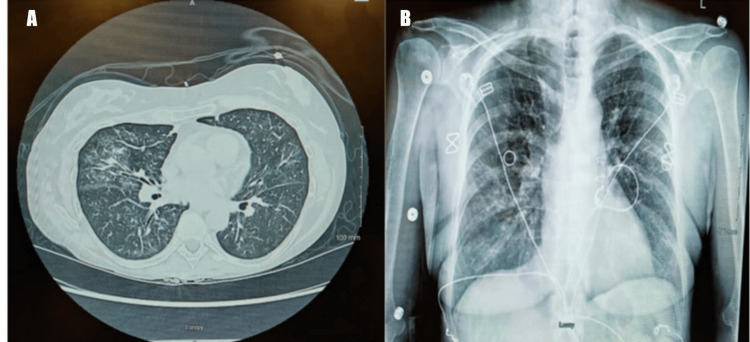
Pulmonary manifestations of anti-MPO-positive GPA: bilateral multifocal ground-glass opacities, tree-in-bud nodules, and patchy alveolar infiltrates demonstrating active vasculitic involvement on CT scan (a) and X-ray (b) GPA, granulomatosis with polyangiitis; MPO, myeloperoxidase

The initial evaluation included a chest X-ray (Figure 2), which demonstrated bilateral patchy alveolar opacities. Given the presence of pedal edema, an echocardiogram was obtained, revealing no cardiac pathology. The patient was subsequently admitted to the internal medicine service. Laboratory evaluation revealed a creatinine level of 11.6 mg/dL and a blood urea nitrogen level of 55 mg/dL. A noncontrast CT scan of the chest demonstrated scattered bilateral multifocal ground-glass opacities with tree-in-bud nodularities (Figure 2), as well as scattered bilateral noncalcified pulmonary nodules, the largest measuring 5 mm.

Bronchoscopy with bronchoalveolar lavage (BAL) was performed to evaluate hemoptysis and exclude diffuse alveolar hemorrhage (DAH) and infectious etiologies. Sequential aliquots were nonhemorrhagic, with no progressively bloody return. BAL analysis demonstrated a nonspecific inflammatory profile without significant red blood cells or hemosiderin-laden macrophages. Microbiologic evaluation, including bacterial, mycobacterial, fungal, and viral studies, was negative, reducing suspicion for infection and DAH.

In the context of the patient’s past medical history and current clinical presentation, a vasculitic flare was suspected. Serologic testing revealed perinuclear ANCA (p-ANCA) titers of 1:80 and an elevated anti-MPO antibody level of 2.3 IU/mL (normal range: 0.0-0.9). Inflammatory markers, including erythrocyte sedimentation rate (ESR) and CRP, were elevated at presentation and demonstrated improvement with treatment. Procalcitonin was not elevated, and blood cultures were negative, further reducing suspicion for bacterial pneumonia. Urinalysis did not reveal active urinary sediment, consistent with her ESRD status.

A biopsy of the painful cutaneous blistering lesions, obtained from the blister edge, demonstrated findings consistent with cutaneous vasculitis, supporting the diagnosis of a GPA flare. She was initiated on IV pulse-dose methylprednisolone at 1 g daily for three days, followed by IV methylprednisolone 40 mg daily for six weeks, with a planned gradual taper over six months. Lesions steadily improved, with reductions in pain, erythema, and drainage. Episodes of hemoptysis became progressively less frequent and eventually resolved, and marked improvement in dyspnea, pedal edema, and fever was also noted. IV rituximab at 375 mg/m² weekly for four weeks was subsequently started to achieve remission, with cyclophosphamide intentionally avoided due to severe renal involvement.

Within six weeks of starting induction therapy with IV methylprednisolone and rituximab, she demonstrated marked improvement. Respiratory symptoms and hemoptysis resolved completely, and the cutaneous lesions showed progressive healing. She is currently on a tapering dose of corticosteroids and continues receiving IV rituximab, reporting steady gains in energy and overall well-being. Follow-up visits occur every three to four weeks in the rheumatology and pulmonology clinics, with routine laboratory monitoring including complete blood count, kidney and liver function tests, ESR, CRP, and urinalysis. Inflammatory markers have steadily normalized, and renal parameters have remained stable. She continues to do well clinically, without evidence of relapse or treatment-related complications, and is expected to transition to maintenance-phase therapy with rituximab (500 mg every six months) in the coming months upon confirmation of sustained remission.

A timeline of events is presented in tabular format in Table 1.

**Table 1:** Timeline of events BAL, bronchoalveolar lavage; DAH, diffuse alveolar hemorrhage; ESRD, end-stage renal disease; MPO, myeloperoxidase; p-ANCA, perinuclear antineutrophil cytoplasmic antibody

Time point	Clinical events
Day 0	Presentation to the ED with progressive dyspnea, hemoptysis, fatigue, subjective fever, and painful cutaneous blistering lesions involving the fingers, elbows, neck, and lower extremities.
Day 1	Chest X-ray demonstrated bilateral patchy alveolar opacities. Initial laboratory evaluation confirmed advanced renal dysfunction consistent with known ESRD.
Day 2	Noncontrast CT of the chest revealed bilateral multifocal ground-glass opacities with tree-in-bud nodules and scattered noncalcified pulmonary nodules. Bronchoscopy with BAL was performed and was negative for DAH and infectious etiologies.
Day 3	Serologic testing showed elevated anti-MPO titers with positive p-ANCA. Dermatology-performed skin biopsy confirmed cutaneous small-vessel vasculitis.
Days 3-5	Induction therapy was initiated with IV pulse-dose corticosteroids.
Week 1	Rituximab induction therapy was commenced.
Week 6	Marked clinical improvement noted, with resolution of hemoptysis, improvement in dyspnea, and healing of cutaneous lesions.

## Discussion

GPA is classically associated with PR3 antibodies, whereas anti-MPO positivity is less common and often overlaps clinically with MPA [3-5]. This case highlights an atypical anti-MPO-positive GPA flare with concurrent pulmonary and cutaneous involvement in a patient with ESRD, illustrating both diagnostic and therapeutic challenges in such presentations. The severity of extrarenal involvement in this patient reinforces the recognized phenotypic overlap between GPA and MPA, particularly in anti-MPO-positive disease [3,4].

Pulmonary manifestations of GPA range from nodules and ground-glass opacities to DAH [5,6]. In this case, hemoptysis and bilateral ground-glass opacities raised concern for DAH; however, BAL was negative. Importantly, the absence of DAH on BAL should not be interpreted as exclusion of active pulmonary vasculitis, as BAL may be falsely negative in early, focal, or partially treated disease and cannot reliably exclude ongoing vasculitic involvement [7]. In pulmonary vasculitis without overt DAH, BAL findings are often nonspecific and may demonstrate a mild to moderate inflammatory profile with increased neutrophils or lymphocytes, absence of progressively hemorrhagic aliquots, and lack of hemosiderin-laden macrophages. In the present case, pulmonary imaging findings, systemic symptoms, elevated anti-MPO titers, and biopsy-proven cutaneous vasculitis collectively supported the diagnosis of an active vasculitic flare despite negative BAL results, underscoring the importance of integrating clinical, radiologic, serologic, and histopathologic assessments.

Management of GPA in patients with established ESRD differs substantially from those with potentially reversible renal involvement, as renal recovery is no longer a therapeutic objective. Treatment is instead directed toward controlling extrarenal disease activity and preventing life-threatening complications. In this context, the risks associated with cyclophosphamide, including infection, myelosuppression, and cumulative toxicity, often outweigh potential benefits, particularly when irreversible renal failure is present [8]. Rituximab, therefore, represents a rational induction strategy, offering comparable efficacy with a more favorable safety profile in high-risk ESRD patients [8,9]. In this case, renal parameters remained stable throughout hospitalization, and urinalysis did not demonstrate active urinary sediment, supporting the interpretation that the flare was driven by systemic vasculitic activity rather than renal relapse. Close coordination with nephrology was essential to ensure dialysis adequacy, monitor volume and electrolyte status, and minimize treatment-related complications during high-dose immunosuppression. Induction therapy with rituximab combined with pulse corticosteroids resulted in rapid resolution of pulmonary and cutaneous manifestations without adverse events, supporting its use as a first-line induction option in ESRD.

In summary, anti-MPO-positive GPA can present with severe multi-system flares, even in patients with advanced renal disease. Negative BAL findings do not exclude active pulmonary vasculitis, and biopsy of accessible lesions remains critical for diagnosis. This case demonstrates that rituximab combined with corticosteroids can be a safe and effective induction strategy in ESRD patients, emphasizing the importance of individualized, multidisciplinary management in atypical GPA presentations and contributing real-world evidence to guide care in this high-risk population [8-10].

## Conclusions

This case highlights the rare but clinically significant presentation of anti-MPO-positive GPA manifesting as a severe multi-organ flare in a patient with ESRD. It underscores that atypical serologic profiles, including p-ANCA positivity with absent PR3 antibodies, do not exclude GPA, and that negative BAL cannot definitively rule out pulmonary vasculitis. The successful use of pulse corticosteroids combined with rituximab demonstrates a safe and effective induction strategy in high-risk patients, providing a viable alternative to cyclophosphamide when renal toxicity or comorbidities limit conventional therapy. This report reinforces the importance of a comprehensive, individualized diagnostic and therapeutic approach in atypical GPA, emphasizes vigilance for multi-system involvement, and contributes to expanding the understanding of phenotypic overlap between GPA and MPA. Early recognition and tailored intervention can achieve rapid remission, even in complex cases with irreversible renal impairment, offering valuable clinical insights for managing rare and challenging vasculitic flares.

In a GPA-like clinical presentation, the absence of PR3 antibodies does not rule out GPA, as anti-MPO antibodies, though rare, can be present in a subset of patients. Negative BAL does not exclude active disease; serology and biopsy remain essential for accurate diagnosis. Pulse corticosteroids combined with rituximab can be considered in patients with severe renal impairment as an alternative to cyclophosphamide.

## Appendices

**Table 2:** Infectious differential diagnoses BAL, bronchoalveolar lavage; IGRA, interferon-gamma release assay

Differential diagnoses	Reason for consideration	Reason for exclusion
Tuberculosis	Subjective fevers, weight loss, and hemoptysis	Ruled out due to negative travel history and IGRA
Invasive aspergillosis	Subjective fevers and hemoptysis	Negative BAL cultures and CT chest findings
Histoplasmosis/blastomycosis/coccidioidomycosis	Concerning radiological and cutaneous findings, along with respiratory symptoms	Negative BAL cultures and negative travel history to fungus-prone areas
Community-acquired pneumonia	Hemoptysis along with fevers and chest X-ray findings	Negative sputum and blood cultures, negative urine streptococcal antigen
Viral bronchiolitis/bronchitis	Cough with blood streaks and rapid progression	Negative BAL fluid viral profile
Lung abscess	History of gradually progressive fevers, weight loss, shortness of breath, and hemoptysis	Negative CT findings and negative sputum cultures
Nocardiosis/actinomycosis	History of gradually progressive fevers, weight loss, shortness of breath, and hemoptysis	Negative CT findings and negative sputum cultures

**Table 3:** Noninfectious differential diagnoses DAH, diffuse alveolar hemorrhage; ESRD, end-stage renal disease; PE, pulmonary embolism

Differential diagnoses	Reason for consideration	Reason for exclusion
Lung malignancy	Chronic cough and hemoptysis with lesions on imaging can raise suspicion for malignancy	CT chest showed multiple bilateral nodules rather than a solitary mass or mediastinal lymphadenopathy. Cytology and bronchoscopy did not reveal malignant cells. No smoking history or weight loss
DAH	Hemoptysis with bilateral diffuse infiltrates and falling hemoglobin can suggest DAH	CT chest findings and bronchoscopy were negative
PE	Hemoptysis with shortness of breath	Negative CT angiogram findings
Coagulopathies	Large-volume hemoptysis and history of ESRD	Normal platelet count and coagulation profile
Upper airway bleed	History of sinus congestion and frontal (sinus zone) headaches	Physical examination and visual inspection of the throat and nose were negative for upper airway bleeding
Left heart failure/mitral stenosis	Shortness of breath, hemoptysis, and mild pedal edema	The echocardiogram showed no evidence of either disease
